# Anxiety, Perceived Stress, and Resilience during the COVID-19 Pandemic: Population Estimates of Persons Presenting to a General Practitioner in Romania

**DOI:** 10.3390/brainsci11111541

**Published:** 2021-11-20

**Authors:** Tiberiu Constantin Ionescu, Bogdana Ioana Fetecau, Voicu Boscaiu, Catalina Tudose

**Affiliations:** 1 Department of Clinical Neurosciences, Faculty of Medicine, University of Medicine and Pharmacy “Carol Davila”, 020021 Bucharest, Romania; catalina.tudose52@gmail.com; 2 Department of Cardio-Thoracic Pathology, Faculty of Medicine, University of Medicine and Pharmacy “Carol Davila”, 020021 Bucharest, Romania; bogdana.fetecau@gmail.com; 3 “Gheorghe Mihoc-Caius Iacob” Institute of Mathematical Statistics and Applied Mathematics, Romanian Academy of Sciences, 050711 Bucharest, Romania; vboscaiu.pr@gmail.com

**Keywords:** anxiety, perceived stress, resilience, COVID-19, pandemic, general practitioner

## Abstract

Facing the COVID-19 pandemic, individuals are experiencing severe mental distress. Thus, during the last year, drastic changes occurred in everyday life of every human being. Following social distancing and economic insecurity, significant increases in mental health concerns (loneliness, anxiety, depression, or insomnia) have developed. The objective of this study was to explore the anxiety, perceived stress, and resilience in a population presenting at the general practitioner, during the COVID-19 pandemic. Data were collected between February and April 2021 and 440 individuals who presented to the general practitioner were evaluated. Concerning anxiety level, almost half of the respondents (49,3%, N = 217) scored above the threshold value on the anxiety scale (mild intensity 38.6%, moderate intensity 9.9%, severe intensity 0.8%). Having a low level of resilience, as well as experiencing a high level of stress, are both predictive of the occurrence of high anxiety (*p* < 0.001, r = −0.551 and *p* < 0.001, r = 0.622, respectively). Furthermore, resilience is negatively related to perceived stress (*p* < 0.001, r = −0.676). It is critical in the current crisis to recognize those at risk of developing mental illnesses, taking into consideration the various socioeconomic classes, as well as to maintain and improve the general public’s mental health using appropriate psychological interventions.

## 1. Introduction

The novel Severe Acute Respiratory Syndrome Coronavirus-2 (SARS-CoV-2) virus spread rapidly to all countries over the world, and the World Health Organization declared an international pandemic on 11 March 2020 [1]. Previous research has demonstrated that infectious disease epidemics are linked to mental health burden among individuals from impacted communities, survivors, and their families [2,3,4]. For example, during the Ebola crisis, a greater number of symptoms associated with posttraumatic stress was observed among residents in impacted communities [5], indicating the need for more attention to these growing symptoms. Although Ebola has a greater mortality rate than Coronavirus disease 2019 (COVID-19), this pandemic has claimed numerous lives worldwide, and research among affected populations has identified risk factors for afflicted people’ mental health [6,7,8,9]. Stress is a significant risk factor for developing a mental disorder (e.g., anxiety and/or depression, somatization, sleeplessness, posttraumatic stress disorder (PTSD), obsessive compulsive disorder (OCD), social phobia, self-harm, and suicidal ideations or behaviors) [7,9,10,11,12,13]. The modes of transmission of SARS-CoV2 and its high contagiousness may be associated with an increased risk of stress characterized by social incapacity and impairment of mechanisms subtending regulation of autonomic responses as a function of environmental stimuli (crowded places, close-contact settings, enclosed spaces). The interpersonal space (IPS) is the area around one’s own body where social interactions normally take place [14]. When other people violate our IPS, such as approaching the subject, emotions of discomfort and even anxiety may arise, triggering a series of physiological responses that manage the distance between ourselves and others during social contact. Ellena et al. confirm the defensive functional definition of IPS by emphasizing how responses to approach stimuli modulate autonomic arousal as a function of the distance between the perceiver and the stimuli, resulting in an appropriate organization of defensive reactions [15]. The use of single-pulse transcranial magnetic stimulation to explore the temporal sequence of the motor system output to relevant arousing stimuli proved to be of great value, reflecting the preparation of adaptive motor responses required for the execution of appropriate and protective behaviors [16]. Moreover, it is worth noting the many other contributing factors for increased stress, like the threat of contracting the virus, infection or death of loved ones, loneliness because of social isolation, quarantine measures, financial instability, and poverty as a result of huge job loss, and last but not least excessive consumption of information from the media.

Regarding anxiety symptoms, the COVID-19 pandemic could be seen as a stressor that elicited a strong anxiety response among people in the epidemic regions [17,18]. A meta-analysis of community-based studies, that included 43 studies, estimated overall prevalence of anxiety of 25% during the COVID-19 outbreak, that could be 3 times higher, comparing to global prevalence of anxiety disorders estimated to be normally around 7.3%. According to the same meta-analysis, the most frequently reported risk factors for the development of anxiety included female gender, younger age, marriage, initial or peak phase of the outbreak, social isolation, poverty, low educational level, unemployment or student status, insufficient knowledge of COVID-19, epidemiological or clinical risk of disease and some lifestyle and personality variables [19,20]. On the other hand, other meta-analyses published in 2020, reported a prevalence of anxiety of 15.15% respectively 31.9%, with significant variations in terms of the main risk factors [10,21]. Therefore, it is very important to understand the psychological factors that predict anxiety in response to such phenomena in order to establish effective preventive mental health care strategies [22].

The present pandemic will affect some more than others, with a highly heterogenous impact on incidence and severity of anxiety disorders. There are quite diverse frameworks when talking about resilience [23], but according to the American Psychological Association, resilience is defined as a “process of adapting well in the face of adversity, trauma, tragedy, threats, or significant sources of stress—such as family and relationship problems, serious health problems, or workplace and financial stressors” [24]. In this study, the resilience concept was used, started from an operational definition of resilience as maintenance or quick recovery of mental health during and after significant stressors. [25,26]. It is essential to focus greater attention on resilience, interpreted as positive individual trajectories of development, to evaluate the potential long-term effects of the COVID-19 pandemic [27,28].

Addressing questions regarding the psychological processes associated with the COVID-19 pandemic, such as the risk of anxiety, depression, or even self-harm and suicide, the impact of media consumption upon mental wellbeing, as well as the protective individual measures for a mentally healthy life, a position paper urged the need for novel population-based studies establishment on mental health and COVID-19, in order to ascertain its potential consequences and the alleged mitigation measures [29]. Nevertheless, another study investigating the potential protective measures for the mental wellbeing during the COVID-19 pandemic, showed that resilience, interpreted as good mental health despite stressor exposure, is positively associated with positive appraisal style, being mediated by the ability to easily recover from stress. The strongest psychological factors proved to be good stress response and positive appraisal specifically of the consequences of the Corona crisis [30].

Shedding light on the role of the general practitioner in the screening of anxiety disorders, given the increasing prevalence of these ailments in the context of the COVID-19 pandemic is of great value. Therefore, by adjusting primary care services to the contemporary socio-cultural background, early detection and management of anxiety disorders will be facilitated. On the other hand, there is still scarcity in the amount of research in Romania on the incidence of anxiety disorders in the context of the COVID-19 pandemic, noteworthy to mention being the fact that the vast majority of these studies were conducted online.

### Pandemic Situation in Romania

**Figure 1:**
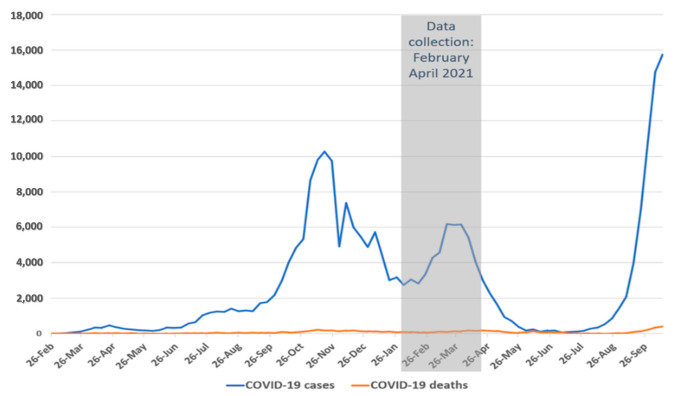
COVID-19 situation in Romania during recruitment. Data from Johns Hopkins University.

The first case of COVID-19 infection in Romania was detected on 26 February 2020 [31] and case numbers have been rising afterwards (see Figure 1), in four waves [32]. Since the beginning of the pandemic, throughout Romania, rigorous national rules became effective, such as the closure of all cultural and educational institutions, reduction in trade and service sectors and finally, restrictions of physical contact and free circulation [33]. All of this and even more rules aimed to inhibit a further exponential growth of the infection numbers. The goal of the present study was to assess anxiety, perceived stress, and resilience regarding the COVID-19 pandemic and to analyze possible protective and risk factors.

## 2. Materials and Methods

This is a cross-sectional study and data were collected between February and April 2021 and 440 individuals who presented to the general practitioner were evaluated. The study sample size was estimated using the Raosoft sample size calculator. A minimum of 340 participants were required at a margin error of 5%, with a 95% confidence interval (CI), for a population size of 2900 (the total number of patients enrolled in the medical office) at a 50% response distribution.

Demographic and medical data were collected, and participants filled in Zung Self-Rating Anxiety Scale (SAS), Connor-Davidson Resilience Scale brief version with 10 items (CD-RISC-10), and Cohen’s Perceived Stress Scale (PSS). 

The study was developed in accordance with the World Health Organization (WHO), the Declaration of Helsinki, European Union legislation, and the ethical principles of clinical research of the Guidelines for Good Clinical Practice (ICH-GCP). All participants received and signed a written consent form before entering the study. The protocol and the informed consent were approved by the Ethical Committee of College of Physicians of Bacau (approval number 46/January 18. 2021).

### 2.1. Participants

*Demographics*—Participant gender (male and female), age (18–24, 25–34, 35–44, 45–54, 55–64, 65–74, and 75 or more), living location (urban and rural), marital status (married, widowed, divorced, in a relationship, and alone), number of children (0, 1, 2, ≥3), level of education (high school graduation, Bachelor’s degree, Master’s degree, Doctoral degree, and postdoctoral studies), occupational status (student, employed, retired, unemployed), and the weekly working hours were assessed by quota sampling. Furthermore, the impact of the COVID-19 pandemic on revenues was evaluated. 

*Comorbidities*—Participants were asked if they have any underlying health conditions (somatic and psychiatric). Those who declared having a somatic or a psychiatric disorder in the previous questions were requested to specify the diagnosis.

*Behavior*—Lifestyle physical activity was evaluated through four levels of intensity: inactive, mild, moderate, and intense. Moreover, tobacco and alcohol consumption habits were evaluated.

*COVID-19 status*, reported for oneself and others, as well as vaccination status were assessed (vaccinated, wanted to get vaccinated, do not want to get vaccinated and the main reason for refusing).

Psychiatric assessment. SAS was used to assess anxiety levels in participants. It contains 20 items that cover the most common symptoms of anxiety, with a ratio between emotional and somatic symptoms of 5/15, this being the reason for which SAS is considered one of the best scales that can identify somatic symptoms [34]. Each symptom is assessed from the subject on a 4-anchor Likert scale based on severity for the last week. The score is reversed for five symptoms (questions 5, 9, 13, 17, and 19). Exploratory factor analysis revealed four lower orders that cover more than 43% of the response options in a sample of people with anxiety: (1) anxiety and panic; (2) vestibular sensations; (3) somatic control; and (4) gastrointestinal/muscular sensations [35]. These factors in turn correlate positively with other scales for measuring anxiety symptoms. The score is calculated by adding the individual score to each subject (raw score). In the present research the threshold value for results interpretation were the following: absence of anxiety < 36, mild intensity anxiety 36–47, moderate intensity anxiety 48–60, severe intensity anxiety > 60. For statistical purposes we also used a related anxiety index to SAS, with threshold values: absence of anxiety < 45, mild intensity anxiety 45–59, moderate intensity anxiety 59–74, and severe intensity anxiety > 75.

The CD-RISC-10 consists of 10 statements describing different aspects of resilience [36]. The scale serves mainly as a measure of robustness, with items corresponding to sense of self-efficacy (items 2, 4, and 9), flexibility (items 1 and 5), optimism (items 3, 6, and 8), cognitive focus/maintaining attention under stress (item 7), and ability to regulate emotion (item 10). Each item is scored on a five-point scale ranging from 0 to 4, with 0 representing that the resilience statement is not at all true and a score of 4 indicating that the statement is true nearly all the time. The total score is obtained by adding up all 10 items. The total can therefore range from 0 to 40. Higher scores suggest greater resilience and lower scores suggest less resilience, or more difficulty in bouncing back from adversity. The scale is neither intended to provide diagnostic information, nor to indicate that treatment or counselling is required. However, in conjunction with other assessments, it could provide one piece of useful information in deciding whether an intervention is appropriate. A score in the lowest or second quartile may suggest problems in coping with stress or hardship.

Perceived stress was assessed with Cohen’s Perceived Stress Scale (PSS) that measured the extent to which participants felt life situations to be stressful [37]. Cohen’s Perceived Stress Scale is a 14-item self-report instrument. Items investigate aspects such as the emotional reaction to negative life situations, the degree of perceived control over sources of stress or the confidence in handling one’s problems [38]. Each item is rated using a 5-point Likert type scale (0 = ‘Never’, 4 = ‘Very Often’), with a total score ranging between 0 and 56. A higher score indicated a more significant presence of perceived stress.

### 2.2. Statistical Analysis 

For data analysis IBM SPSS version 22 was used. The association between qualitative variables used Pearson chi-square test and Fisher’s exact test. One-way ANOVA parametric test, Kruskal–Wallis, and median non-parametric tests were considered for multiple comparison of quantitative variables. For all analyses, the level of statistical significance was set at *p* < 0.05.

## 3. Results

### 3.1. Sociodemographic Characteristics

**Figure 2:**
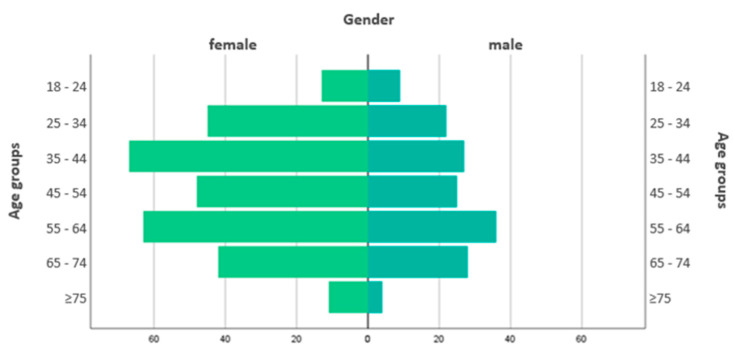
Pyramid of age groups by gender. N = 440, 65.7% female (N = 289); the most representative age group was 35–64 years old (60.5%, N = 266).

The final sample consisted of 440 participants, 65.7% female (N = 289) and the most representative age group was 35–64 years old (Figure 2); three quarters were married or in a stable relationship (75.7%, N = 333), living with a partner (70.7%, N = 311) (Table 1). Most participants were employed (53.4%, N = 235) and for almost half of the subjects their revenues decreased (44.8%, N = 197).

About 53.4% of cases (N = 235) suffered from a pre-existing physical illness, mainly cardiovascular diseases (34.1%), rheumatic or musculoskeletal conditions (12.5%), as well as diabetic and metabolic disorders (11.8%). Regarding mental disorder, 7.0% (N = 31) reported to have a pre-existing diagnostic, more frequently being major depressive and anxiety disorders (Table 2).

Most respondents stated that they do regular mild or moderate intensity exercise (70.5%, N = 310) (Table 3). Regarding tobacco consumption, 74.1% of the respondents did not smoke, of these, 44.3% had never smoked. Regarding alcohol consumption, 48.6% reported that they never drink alcohol and 50.0% reported that they drink less than 2 units/day. The smoking and drinking habits have decreased during the last year; 11.4% of the respondents reported that they decreased the number of cigarettes smoked per day, while 48.6% of the sample reduced the alcohol consumption, after the pandemic was declared.

### 3.2. COVID-19 Status

Regarding SARS-CoV-2 infection, 18.4% (intensity of symptoms: asymptomatic 9.9%, mild 30.9%, moderate 53.1%, severe 6.2%) of the participants stated that they were infected themselves, 44.5 % reported knowing people with an infection in their close social environment (family or friends), and 10.5% had close people who died of COVID-19. At the time the study was conducted, 18.9% of respondents were vaccinated and 41.6% wanted to get vaccinated, however 35.2% of the sample did not want to get vaccinated and the main reason for refusing to take the vaccine was the lack of trust in the vaccines (84.1%). 

**Figure 3:**
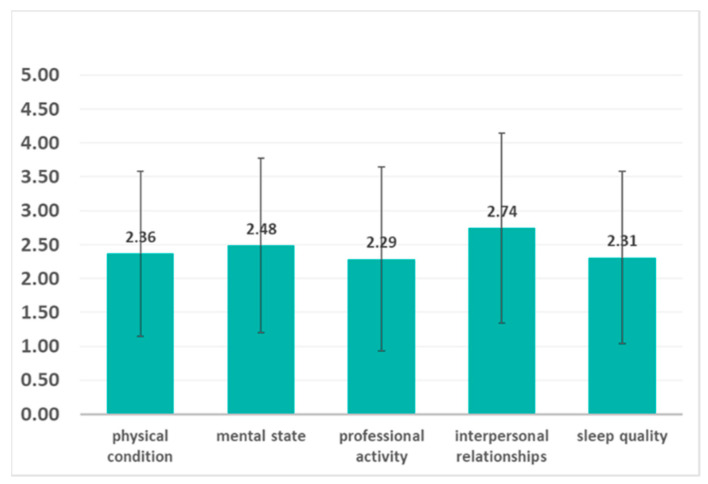
The impact of the pandemic on functionality. Using 5-point Likert scales (1 means not at all, and 5 means completely affected), functioning was evaluated in five areas. Interpersonal relationships (mean = 2.74, SD = 1.40) and mental state (mean = 2.48, SD = 1.28) were the most affected by the pandemic, followed by impaired physical condition (mean = 2.36, SD = 1.21), sleep quality (mean = 2.31, SD = 1.26), and professional/academic activities (mean = 2.29, SD = 1.36).

The questionnaire contained 5 questions that assessed how the pandemic affected the physical condition, the mental state, the professional activity, interpersonal relationships, and the quality of sleep. Respondents were asked to respond using a 5-point Likert scale, where 1 means not at all, and 5 means completely affected. Average scores and standard deviations are presented in Figure 3.

The main concerns associated with the pandemic context were evaluated through a multiple-choice question. COVID-19 stress related factors are fear of illness/death of close people (57.3%, N = 252), concerns regarding the capacity of the health system (35,7%, N = 157), social isolation (34.1%, N = 150), economic impact (31.1%, N = 137), fear of illness/death (30.5%, N = 134), fear that the basic protective measures (mask, disinfection of hands) are not enough (16.8%, N = 74), concerns regarding the risk of becoming unemployed (14.8%, N = 65), as well as social stigma associated with COVID-19 infection (8.6%, N = 38). The major source of information on COVID-19 was the traditional media, including television, newspapers, and radio broadcasting (63.20%), and from the medical staff in health care settings (51.80%), followed by Internet including blogs, news websites, and social media (37.70%), and friends, co-workers, and family members (22.70%).

### 3.3. Clinical Characteristics

**Figure 4:**
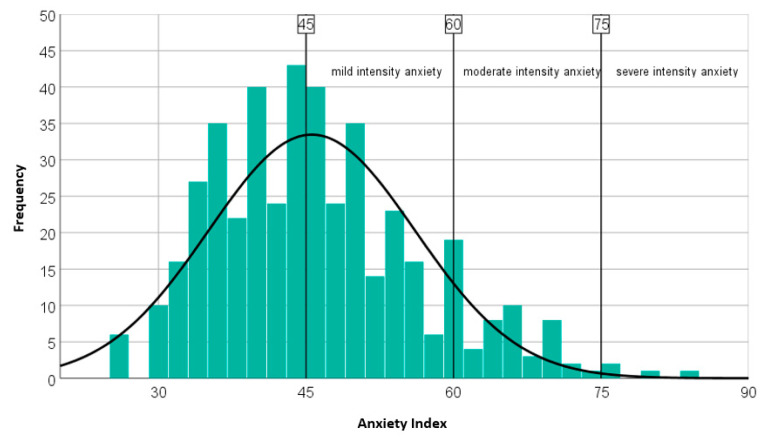
Level of anxiety on the SAS. In total, 49.3% of the respondents (N = 217) reported high levels of anxiety: mild intensity 38.6% (N = 170), moderate intensity 9.9% (N = 43), severe intensity 0.8% (N = 4).

Concerning anxiety level, almost half of the respondents (49.3%) scored above the threshold value on the SAS (mild intensity 38.6%, moderate intensity 9.9%, severe intensity 0.8%) (Figure 4), with a mean anxiety index and a standard deviation (SD) of 45.58 and 10.49, respectively. In particular, the female participants scored significantly higher compared to male (mean female anxiety index = 46.85, mean male anxiety index = 43.16, *p* < 0.001) and respondents under 25 years old and those 65 years of age or older are more likely to experience symptoms of anxiety (*p* < 0.001). 

**Figure 5:**
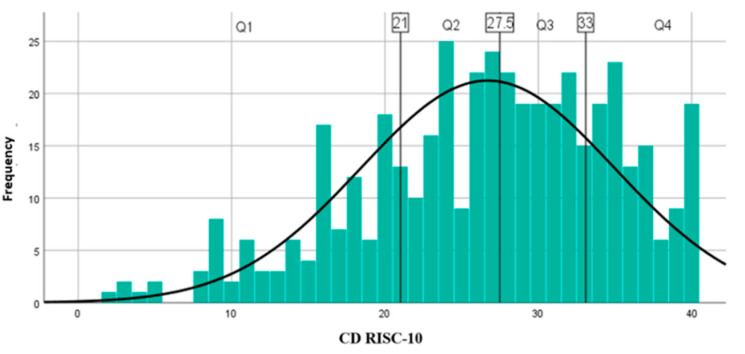
Resilience level. Q1 = lowest quartile (1 to 25%) scored between 0 and 21; Q2 = the second quartile (26 to 50%) scored between 21 and 27.5; Q3 = the third quartile (51–75%) scored between 27.5 and 33; Q4 = the top quartile (76–100%) scored between 33 and 40.

The level of resilience during pandemic was calculated based on the CD–RISC–10 scores and ranged from 2 to 40 among all participants (mean: 26.70 ± 8.26) (Figure 5), from 3 to 40 among female (mean: 26.55 ± 8.37), and from 2 to 40 among male (mean: 26.98 ± 8.08). In the female groups the median score (Q25–Q75) on the CD-RISC-10 was 27 (range 21–27), while in the male subset the median score (Q25–Q75) on the CD-RISC-10 was 28 (range 28–33). Gender was not significantly associated with resilience level (*p* = 0.608). Higher resilience was associated with age between 25 and 54 years old (*p* < 0.001) (Table 4).

**Figure 6:**
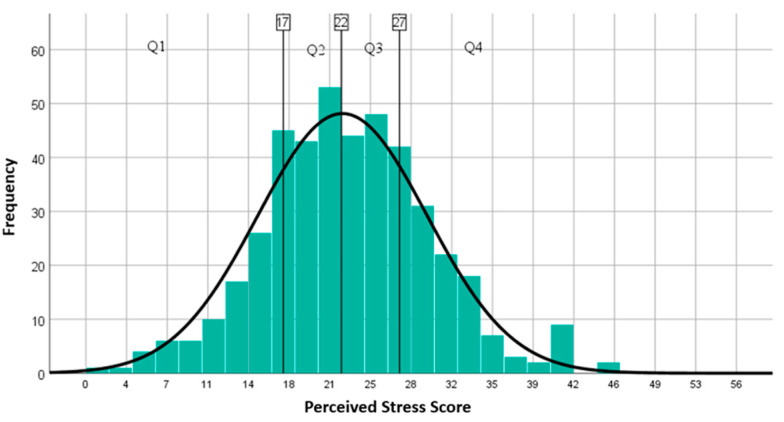
Perceived Stress Level. Q1 = lowest quartile (1 to 25%) scored between 1 and 17; Q2 = the second quartile (26 to 50%) scored between 18 and 22; Q3 = the third quartile (51–75%) scored between and 33; Q4 = the top quartile (76–100%) scored between 33 and 40.

The PSS-14 score in the study group ranged from 1 to 44 and the mean score (Q25–Q75) was 22.09 (SD = 7.292) (Figure 6). No significant correlation was found between gender and perceived stress (PSS-14 female mean score = 22.50 with SD = 7.126 and male mean score = 21.30 with SD = 7.561), *p* = 0.708. Higher perceived stress levels were reported at respondents under 25 years old and over 55 years old (Table 4).

Respondents living in the urban areas reported higher stress levels (*p* = 0.011) and lower resilience (*p* = 0.008) than respondents from the countryside. Lower level of education is statistically significant correlated with anxiety symptoms, perceived stress, and low resilience. Furthermore, unemployed and retired respondents associated higher anxiety and lower resilience. Based on expectations, study participants whose incomes have declined in the current pandemic context, associated an increase in anxiety and perceived stress levels, as well as decreased resilience. Regarding weekly working hours, respondents who work less than 20 h per week report the highest levels of anxiety and stress, as well as the lowest levels of resilience, even though we would expect a busy work schedule to be associated with these feelings. (Table 5).

Concerning the respondents’ primary worries about the pandemic, fear of sickness or death of individuals in their immediate circle was associated with elevated levels of anxiety (*p* < 0.001) and perceived stress (*p* = 0.001), as well as a low degree of resilience (*p* = 0.011). Furthermore, a low degree of resilience was associated with the fear of disease and death (*p* < 0.001). Concern of stigmatization by others in the event of infection was associated with higher levels of anxiety (*p* = 0.013).

Use of the traditional media as a primary source of information regarding COVID-19 was significantly associated with higher anxiety levels (mean score: 46.67 compared to 43.72; *p* < 0.001), higher perceived stress (mean score: 22.81 compared to 20.85; *p* = 0.006), and lower levels of resilience (mean score: 25.83 compared to 28.20; *p* = 0.004). 

## 4. Discussion

As a result of the current COVID-19 outbreak, there has been an understandable increase in worry among the wider population. Having a low level of resilience, as well as experiencing a high level of stress, are both predictive of the occurrence of high anxiety when someone close to you dies. Furthermore, resilience is negatively related to perceived stress (Table 6). In the study population, the severity of anxiety symptoms is not associated with COVID-19 infection status, the intensity of symptoms, or the amount of time that has passed since infection.

Mortality owing to COVID-19 has been linked to advanced age and concomitant chronic illnesses, which have been identified as the most significant risk factors [39,40]. In addition, the elderly and those suffering from chronic illnesses are at a higher risk of acquiring the condition [41]. Individuals with chronic illnesses have higher levels of anxiety (cardiovascular diseases, *p* < 0.001; metabolic conditions, *p* < 0.001; pulmonary conditions, *p* = 0.020; gastrointestinal conditions, *p* < 0.001), stress (cardiovascular diseases, *p* < 0.001; gastrointestinal conditions, *p* = 0.021), and poor resilience (cardiovascular diseases, *p* < 0.001; metabolic conditions, *p* = 0.037; gastrointestinal conditions, *p* = 0.024), which are all results that run parallel to one another. 

People who have pre-existing mental disorders, as has been shown in previous research [42], are more likely to have difficulties in managing stress (*p* < 0.001) during the pandemic and to have a substantially poorer coping ability than those without a pshychiatric illnesses (*p* < 0.001). Action control deficits were observed in people with pre-existing psychiatric disorders like anxiety and depression, OCD, or conduct disorder [43]. This means that they are predisposed to coping mechanisms that are ineffective in the face of chronic stress, such as a context-specific pandemic. The association between altered neural pathways and pathological states such as trauma, stress, or anxiety disorders which lead to poor coping strategies, has also been examined in several studies. Fear-memory reconsolidation is influenced by several neural networks, and these connections might be used to treat emotional or maladaptive memories in the future. The role of dorsolateral prefrontal cortex memory reactivation in disrupting physiological responses to learned fear is already established. More interestingly, there are promising therapeutic methods of non-invasive brain stimulation (NIBS), as valid alternatives in the treatment of abnormally persistent memories for those patients with anxiety disorders who do not respond to psychotherapy and/or drug treatments [44,45]. Fear processing has been improved, but new technologies like NIBS and optogenetics will lead to a more specific identification and manipulation of the neurobiology of fear, which might lead to the discovery of a suitable therapeutic target for pathological fear states [46]. This fear-inducing factor, the COVID-19 pandemic, activates specific neural networks, which in turn determine dysfunctional behaviors, such as lack of action control and motor inhibition, being associated with psychopathological and psychiatric conditions characterized by serious impulsivity problems that could determine severe impairment or distress [47].

For statistical purposes, the sample was divided in three groups: 56.6% reported that they do not know anyone in their entourage who was infected/died of COVID-19 (group A), 32.9% knowing people with SARS-CoV2 infection in their close social environment (group B), and 10.5% had close people who died (group C). If groups A and B had similarities regarding anxiety levels (44.97 and 44.23), those knowing someone who died of COVID-19 (group C) had a higher anxiety level (47.81%), *p* = 0.05. Knowing a loved one with a confirmed COVID-19 case was not linked to an increased incidence of anxiety, contrary to previous research [48,49,50]. Having a loved one with a confirmed case of COVID-19 or a loved one deceased of COVID-19 were not associated with increased perceived stress level or decreased resilience level. 

Except for investigations involving healthcare professionals, this is one of the first studies to investigate the prevalence of anxiety in Romania, as well as the relationship between anxiety and perceived stress and resilience. Nearly 40% of respondents reported a mild level of anxiety, while over 10% reported scores indicating moderate to severe symptoms. Prevalence of anxiety among respondents is higher compared to other studies [10,21,51]. For instance, a multicenter study, conducted in four countries in western and northern Europe, reported anxiety rates between 20% and 30%, with a slight downward trend by mid-2020 [52].

Finally, major predictors of higher anxiety related to the pandemic outbreak included demographic factors, like being female, being younger than 25 and older than 65, being unemployed or retired, having a low level of education, and having decreased revenue. These demographic variables were also associated with positive screening for GAD or depression in other online surveys during this pandemic [48,50]. Additionally, greater anxiety was reported among people who reported chronic illnesses or mental disorders. From COVID-19 related factors, fear of sickness or death of individuals in their immediate circle and fear of stigmatization by others in the event of infection, as well as use of the traditional media as a primary source of information regarding COVID-19 were associated with higher levels of anxiety.

### Limitations

There were some limitations to this study. First and foremost, because the samples were obtained from only one general practitioner in Romania, it is prudent not to assume the extension of the results worldwide. Secondly, the results were only investigated at a specific point in time. This means that the findings of the study are simply a “snap-shot” of the mental health of the individuals who presented to their primary care physician for treatment. The third limitation is that we did not examine all the factors that could have an impact on the participants’ levels of anxiety, stress, and resilience. More in-depth and prospective studies on anxiety, stress, and resilience will be required in the future. As a fourth point, due to the cross-sectional study design, the causal correlations between the variables should be interpreted with care. It is advisable that further research should be conducted to confirm the validity of the current findings.

## 5. Conclusions

During the last year, the COVID-19 pandemic has triggered a global state of emergency. According to our findings, patients who attended their general practitioner had a high level of anxiety, which was linked to a variety of predictors, including gender and age, the presence of psychiatric or somatic comorbid conditions, along with socioeconomic and occupational status. Thus, in the current crisis, it is critical to identify individuals predisposed to anxiety disorders across diverse groups and layers of populations. An established screening method for anxiety in the primary care units and the development of a guideline dedicated to general practitioners on the recommendations of psychotherapeutic and psychopharmacological interventions, including telemedicine, is mandatory. Leveraging these possibilities is essential for managing the increased mental health treatment demands emerging as a result of the COVID-19 pandemic and minimizing existing disparities in accessing evidence-based mental health care.

## Figures and Tables

**Table 1:** Demographic characteristics.

Place of Residence, % (N)		
Urban	84.5	(372)
Rural	15.5	(68)
**Marital Status, %, (N)**		
Married	297	(67.5)
Widowed	38	(8.6)
Divorced	27	(6.1)
In a relationship	36	(8.2)
Alone	42	(9.5)
**Number of Children, %, (N)**		
0	21.4	(94)
1	32.5	(143)
2	38.2	(168)
≥3	8.0	(35)
**Living with Someone Else, %, (N) 84.5 (372)**		
**Education Level, %, (N)**		
High school graduation	54.1	(238)
Bachelor’s Degree Certificate	30.9	(136)
Master’s Degree Certificate	12.5	(55)
Doctoral Degree Certificate and Postdoctoral studies	2.5	(11)
**Occupation, %, (N)**		
Unemployed	8.4	(37)
Student	3.0	(13)
Employed	53.4	(235)
Retired	35.2	155)
**Revenues, %, (N)**		
Decreased	44.8	(197)
Stagnated	38.4	(169)
Increased	16.8	(74)
Weekly Working Hours *, %, (N)		
<20	45.7	(201)
20–39	21.6	(95)
>40	32.7	(144)

Note. Values are depicted as percentages and numerical data. * Does not include time spent by students studying online/on campus.

**Table 2:** Comorbidities.

Any Comorbid Mental Condition(s) %, (N)		
Yes	7.0	(31)
No	93.0	(409)
**Mental Condition(s), %, (N)**		
Major depression disorder	4.8	(21)
Anxiety disorder	0.7	(3)
Others	1.4	(6)
**Any Comorbid Physical Condition(s)**		
Yes	53.4	(235)
No	46.6	(205)
**Physical Condition(s), %, (N)**		
Cardiovascular	34.1	(150)
Rheumatic or musculoskeletal	12.5	(55)
Metabolic	11.8	(52)
Gastrointestinal	7.0	(31)
Pulmonary	5.2	(23)
Oncologic	5.2	(23)
Renal	4.5	(20)
Other	4.7	(21)

Note: Values are depicted as percentages and numerical data. Comorbidities are not mutually exclusive. A total of 104 participants have two or more physical comorbidities.

**Table 3:** Physical activity and smoking and drinking habits.

Physical Activity, %, (N)					
Inactive	22.0			(97)	
Mild	40.7			(179)	
Moderate	29.8			(131)	
Intense	7.5			(33)	
**Use of Alcohol and Tobacco**					
**Smoking Status, %, (N)**			**Drinking Status, %, (N)**		
Never smoked	44.3	(195)	Never drank	48.6	(214)
Former smoker	29.8	(131)	<2 units/day	50	(220)
<20 cigarettes/day	24.8	(109)	>2 units/day	1.4	(6)
>20 cigarettes/day	1.1	(5)			
**Smoking Habit in the Pandemic, %, (N)**			**Drinking Habit in the Pandemic, %, (N)**		
Decreased	11.4	(50)	Decreased	31.8	(140)
Stagnated	84.3 *	(371)	Stagnated *	65.2	(287)
Increased	4.3	(19)	Increased	3.0	(13)

Note. Values are depicted as percentages and numerical data. * Includes those who did not smoke or drink alcohol, nor before the pandemic, nor when the study was conducted.

**Table 4:** Resilience and perceived stress levels among different age groups.

Resilience among Different Age Groups		CD RISC-10 ^a^				
		Mean	Standard Deviation	Median	Percentile 25	Percentile 75
**Age Groups**	18–24	26.55	5.90	27	24.00	30.00
	25–34	28.69	7.78	29	23.00	34.00
	35 = 44	28.36	7.83	30	25.00	34.00
	45–54	28.21	7.72	29	24.00	34.00
	55–64	25.82	8.31	26	20.00	33.00
	65–74	23.87	8.78	24	18.00	31.00
	>75	19.33	8.62	18	11.00	27.00
**Stress among Different Age Groups**		**PSS-14 ^b^**				
		**Mean**	**Standard Deviation**	**Median**	**Percentile 25**	**Percentile 75**
**Age Groups**	18–24	23.14	7.56	23.00	18.00	28.00
	25–34	19.40	8.25	19.00	14.00	25.00
	35 = 44	20.70	6.82	21.00	16.00	25.00
	45–54	21.63	6.68	20.00	17.00	24.00
	55–64	22.58	7.00	22.00	17.00	27.00
	65–74	24.84	6.71	25.00	20.00	29.00
	>75	27.40	6.15	27.00	22.00	31.00

Note. CD RISC-10 = Connor-Davidson Resilience Scale brief version with 10 items; PSS-14 = Cohen’s Perceived Stress Scale; ^a^. Range is 0–40, a higher score indicates greater resilience; ^b^. Range is 0–56, a higher score indicates a more significant presence of perceived stress.

**Table 5:** Correlation between demographic characteristic and scale scores.

Variables	Anxiety SAS ^a^, Mean Score (SD)	*p* Value	Resilience CD RISC-10 ^b^ Mean Score (SD)	*p* Value	Stress PSS ^c^ Mean Score (SD)	*p* Value
**Education Level**						
High school graduation	52.31 (+/−10.34)	<0.001 **	20.79 (+/−9.42)	<0.001 **	26.44 (+/−8.43)	<0.001 **
Bachelor’s Degree Certificate	44.35 (+/−9.08)		27.16 (+/−6.80)		21.95 (+/−6.82)	
Master’s Degree Certificate	43.85 (+/−11.04)		29.24 (+/−7.55)		20.87 (+/−6.50)	
Doctoral Degree Certificate	37.18 (+/−5.72)		33.82 (+/−3.74)		15.64 (+/−6.04)	
**Occupation**						
Unemployed	49.26 (+/−12.59)	=0.009 **	23.29 (+/−9.90)	=0.046 *	25.94 (+/−8.74)	=0.9
Student	43.77 (+/−6.88)		28.46 (+/−5.77)		21.92 (+/−7.03)	
Employed	42.47 (+/−9.70)		28.80 (+/−7.00)		20.00 (+/−6.64)	
Retired	48.75 (+/−10.40)		24.21 (+/−8.87)		24.15 (+/−6.91)	
**Revenues**						
Decreased	47.74 (+/−10.51)	<0.001 **	25.38 (+/−8.91)	<0.001 **	24.69 (+/−7.71)	=0.001**
Stagnated	44.56 (+/−9.98)		27.11 (+/−7.77)		21.80 (+/−6.96)	
Increased	39.47 (+/−8.48)		29.25 (+/−5.64)		20.49 (+/−5.81)	
Weekly Working Hours ***						
<20	48.46 (+/−10.94)	<0.001 **	23.79 (+/−9.10)	<0.001 **	24.11 (+/−7.80)	<0.001 **
20–39	42.27 (+/−8.18)		28.15 (+/−7.23)		20.67 (+/−5.81)	
>40	43.78 (+/−9.67)		29.40 (+/−6.12)		20.36 (+/− 6.58)	

Note. SAS = Zung Self-Rating Anxiety Scale; CD RISC-10 = Connor-Davidson Resilience Scale brief version with 10 items; PSS = Cohen’s Perceived Stress Scale; ^a^. Anxiety index range from 0 to 100 and the threshold values: absence of anxiety <45, mild intensity anxiety 45–59, moderate intensity anxiety 59–74 and severe intensity anxiety > 75, ^b^. Range is 0–40, a higher score indicates greater resilience; ^c^. Range is 0–56, a higher score indicates a more significant presence of perceived stress. * Correlation is significant at the 0.05 level (2-tailed). ** Correlation is significant at the 0.01 level (2-tailed). *** Does not include time spent by students studying online/on campus.

**Table 6:** Interscale correlation.

		Resilience Level (CD RISC-10)	Stress Level (PSS)	Anxiety Index (SAS)
**Resilience Level** **(CD RISC-10)**	Pearson Correlation	1	−0.676 **	−0.551 **
	Sig. (2-tailed)		0.000	0.000
**Stress Level** **(PSS)**	Pearson Correlation	−0.676 **	1	0.622 **
	Sig. (2-tailed)	0.000		0.000
**Anxiety Index** **(SAS)**	Pearson Correlation	−0.551 **	0.622 **	1
	Sig. (2-tailed)	0.000	0.000	

Note. SAS = Zung Self-Rating Anxiety Scale; CD RISC-10 = Connor-Davidson Resilience Scale brief version with 10 items; PSS = Cohen’s Perceived Stress Scale. **. Correlation is significant at the 0.01 level (2-tailed).

## Data Availability

The data presented in this study are openly available in Mendeley Data at doi:10.17632/c9x7gj6c8p.1, reference number: [53].
